# Fast and Selective Plasmonic Serotonin Detection with Aptamer-Gold Nanoparticle Conjugates

**DOI:** 10.3390/s17040681

**Published:** 2017-03-25

**Authors:** Jorge L. Chávez, Joshua A. Hagen, Nancy Kelley-Loughnane

**Affiliations:** 711th Human Performance Wing, Airman Systems Directorate, Air Force Research Laboratory, Wright-Patterson Air Force Base, Dayton, OH 45433, USA; jorge.chavez_benavides.2@us.af.mil (J.L.C.); joshua.hagen.1@us.af.mil (J.A.H.)

**Keywords:** serotonin detection, aptamer, colorimetric, gold nanoparticles, biofluids

## Abstract

Neurotransmitters detection is critical to understanding communication between the brain and peripheral tissue. Serotonin is a key neurotransmitter linked to a number of conditions, but a full understanding of its role in disease is still lacking. The development of fast and selective serotonin detection platforms will provide researchers with tools to monitor serotonin in individuals before and after treatment for the condition of interest. Aptamer-gold nanoparticles conjugates that responded colorimetrically to serotonin with minimal response to its metabolite and other neurotransmitters were designed by simply adsorbing the DNA on the surface of AuNPs. A plasmonic assay for serotonin detection was designed with a response to biologically relevant serotonin levels. Importantly, the assay performance was not compromised when tested in filtered spiked fetal bovine serum as a mimic of biofluids. This work shows that these simple and stable Apt-AuNP conjugates are promising tools to develop fast assays for point-of-care and personalized diagnostics applications.

## 1. Introduction

Serotonin (5-hydroxytryptamine) is a neurotransmitter of critical importance in the central nervous system with neuromodulation properties influencing sleep pattern regulation, aggression and drive, appetite control, and sexual activity [1]. Serotonin is synthesized in different locations including the brain, spinal cord, and the intestine [2] and exists as free-serotonin or serotonin contained in platelets. Serotonin is found in the circulatory system, which is where it was originally discovered [3,4]. Although its physiological role is not fully understood [2], peripheral serotonin levels and their imbalance have been linked to a number of conditions including primary pulmonary hypertension [5], blood pressure regulation [6], kidney disease [7], and depression [8]. While platelet-poor plasma serotonin levels are in the ~1–100 nM range [9], typical serotonin levels in whole blood have been reported to be in a range that spans from ~500 nM to ~1.7 µM in healthy subjects [10]. Importantly, patients with major depressive disorder were found to have lower serotonin blood levels than healthy controls (~0.3 µM vs. ~1.15 µM) [8] and a similar trend was observed for type 2 diabetes with chronic kidney disease (~0.5 µM vs. ~0.713 µM) [7]. These results show the potential of using serotonin as a biomarker for identifying different conditions and for monitoring treatment to restore its imbalance.

Multiple methods to quantify serotonin in whole blood and platelet-poor plasma have been proposed. Most of these methods are based on HPLC separation of serotonin and other species with similar chemical structures present in whole blood, coupled to a detection method such as MS-MS [3] or measurement of its intrinsic fluorescence [7,8,11]. These approaches have provided a number of platforms for accurate and precise serotonin quantification, but they have a few drawbacks that prevent their implementation in rapid diagnostics formats: they require specialized instrumentation and are time consuming. Therefore, tools for monitoring serotonin levels in whole blood that are fast, use low samples volumes, and require minimal sample treatment would be of great value in the clinical field, especially in the emerging field of personalized medicine.

Recently, new sensor platforms for serotonin detection have been proposed, including modified electrodes [12,13,14], cyclodextrins [15], and molecularly imprinted polymers [16,17]. Aptamers are an appealing alternative for creating sensing approaches for catecholamines and other neurotransmitters. Aptamers are versatile DNA or RNA capture elements that can be selected to bind an analyte with great selectivity over chemically similar species [18,19]. They offer many advantages over other biorecognition elements, including their ease of chemical derivatization for sensor immobilization, thermal stability, and structure switching properties that are advantageous for transducing the binding event into different outputs in a sensor platform [20]. The combination of gold nanoparticles and aptamers has resulted in the design of a number of successful colorimetric sensors for different targets that have revolutionized the field of diagnostics [21,22]. In this contribution, we report the design of an assay for serotonin based on the use aptamer-gold nanoparticle conjugates (Apt-AuNPs) that utilizes small sample volumes (10 µL), with short response times (<15 min) and with an easily detectable output (colorimetric) that could be coupled to portable devices such as smartphones and tablets for on-the-spot analysis [23].

## 2. Materials and Methods

All chemicals were obtained from Sigma-Aldrich (St. Louis, MO, USA), the HPLC-purified DNA aptamer (catalog number: Nore-5XO2O293932C) was purchased from Base Pair Biotechnologies (Pearland, TX, USA), and methanol was obtained from OmniSolv (Charlotte, NC, USA). Amicon filters with 3 kDa MWCO, PBS buffer (10×), nuclease-free water, and fetal bovine serum were purchased from Invitrogen. AuNPs were home-made and synthesized as reported previously with no modifications [24]. The AuNP extinction was measured in a Synergy HT plate-reader from Biotek (North Shoreline, WA, USA).

### 2.1. Apt-AuNPs Preparation

The DNA was introduced to the “as-prepared” AuNPs at a ratio of 150 strands of DNA per AuNP, and allowed to incubate for two hours. Typically, 2.5 mL of a 10 nM AuNP suspension was mixed with 4.5 µL of a 1 mM aptamer solution prepared in DNAse-free water followed by vortexing for 1 min. Subsequently, the Apt-AuNPs were mixed with an equal volume of a 0.25× PBS buffer supplemented with 2 mM·MgCl_2_ (pH 7.4), to obtain a final buffer composition in the system of 0.125× PBS/1 mM·MgCl_2_ (assay buffer). After overnight incubation, the Apt-AuNPs were ready to be used for serotonin detection. A similar protocol was followed to prepare the negative control Apt-AuNPs, using an estradiol-binding aptamer [25].

### 2.2. Transmission Electron Microscopy

Images were taken in a Hitachi H-7600 (Hitachi Ltd., Tokyo, Japan), samples were prepared as described in Section 2.3. The Apt-AuNPs were exposed to serotonin (5 µM) followed by NaCl addition. After 15 min of incubation, a drop of the suspension was deposited in a 200 mesh copper grid with a formvar carbon film (Electron Microscopy Sciences, Hatfield, PA, USA). Fifteen minutes later, the top liquid was removed and left to dry overnight before imaging was performed. To avoid over-aggregation due to drying, the NaCl concentration was reduced to half its typical value (250 mM).

### 2.3. Serotonin Assay

Serotonin solutions were prepared by diluting stock methanolic solutions 10 times with an assay buffer to obtain the serotonin concentrations needed. Having a fixed dilution factor for all stock solution helped to keep the methanol content consistent and avoid any effects from varying amounts of organic solvent in the assay response. To test the response of the Apt-AuNPs, 90 µL of Apt-AuNPs were mixed with 10 µL of the serotonin solution and incubated for 5 min, which was followed by an addition of a stock solution of NaCl. The assay was optimized by finding the NaCl concentration that promoted severe Apt-AuNP aggregation when exposed to a blank sample (buffer containing a 10% methanol) but moderate or minimal aggregation in the presence of serotonin. The aggregation degree was monitored for 7 min. In this work, we defined the aggregation degree as the ratio of the AuNPs extinction at 530 nm (dispersed AuNPs) and 650 nm (aggregated AuNPs). The assay was further tuned for maximum response by maximizing the difference between the signal obtained with the blank and a 1 µM serotonin solution. The assay test in biofluids was performed with FBS that was filtered first with a 0.22 µm sterile nylon filter, followed by centrifugation of the filtered FBS with 3 kDa MWCO Amicon filters. After 10 min, the supernatant was cloudy and retained most of the larger components, while leaving the centrifugate clear. The treated FBS was then spiked with different serotonin and epinephrine concentrations, keeping the same FBS content. The assay was re-adjusted as explained above to obtain the salt concentration necessary to allow a clear difference in response to the target and negative controls.

## 3. Results

**Figure 1:**
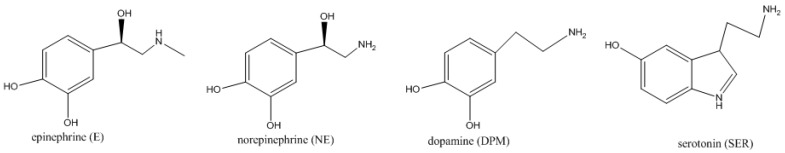
Chemical structure of neurotransmitters used in this study.

Citrate-stabilized AuNPs (~15 nm) have been used in different sensor platforms due to their ability to interact with different species and facilitate signal generation. For instance, different molecules and proteins can coordinate with the AuNPs’ surfaces, resulting in a layer of adsorbed species that affect the AuNPs’ stability, either increasing or decreasing their susceptibility to aggregation in the presence of salt [26]. However, the relatively fragile nature of citrate-stabilized AuNPs might limit their application in neurotransmitter detection in complex biofluids such as blood or serum, due to the presence of multiple chemical species, including proteins with high surface activity that tend to form a protein corona, see Figure 1 for chemical structures of neurotransmitters of interest in this study [27]. Therefore, we decided to tune the selectivity of the AuNPs toward serotonin by using aptamers, as shown below.

### 3.1. Characterization of Apt-AuNPs’ Response to Serotonin

**Figure 2:**
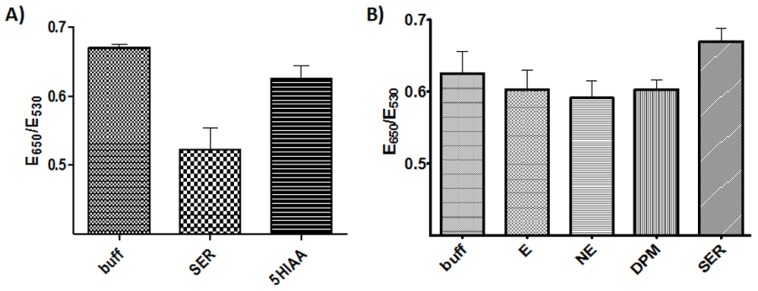
Assay characterization. (**A**) Apt-AuNP aggregation degree after exposure to a blank, serotonin, and 5-HIAA (5 µM each) 2 min after the addition of NaCl (484 mM). (**B**) Assay performed with estradiol-binding aptamer-coated AuNPs, using analytes at a concentration of 100 µM and NaCl of 750 mM, E = epinephrine, NE = norepinephrine, DPM = dopamine.

The serotonin sensing Apt-AuNPs were prepared by the physical adsorption of the DNA on the AuNPs’ surfaces through an overnight incubation in a buffer. In this step, the DNA is left to interact at room temperature with the AuNPs and a stable conjugate is obtained that remains well dispersed with minimal aggregation for a few weeks, stored at 4 °C. We have previously demonstrated that this type of Apt-AuNP could be designed with different aptamers that target different small molecule analytes [23,28,29]. Our first experiments demonstrated that the binding of serotonin to Apt-AuNPs improved their stability against salt-induced aggregation, as shown in Figure 2A. Importantly, in most cases of aptamer-controlled AuNP-based assays, analyte binding resulted in AuNP aggregation due to a target-induced aptamer conformational change that disrupted the stabilizing interactions provided by the negatively charged DNA strands [30]. In the case observed here, as shown in Figure 2A, the aggregation degree decreased in the presence of serotonin, compared to the blank and to its metabolite 5-hydroxyindoleacetic acid (5-HIAA). To investigate whether this response was mediated by the DNA on the AuNPs’ surfaces, we tested the assay in a similar fashion using an estradiol-binding aptamer that our group has used in the past [25]. In this case, the NaCl needed was adjusted to result in an aggregation degree similar to the blank so that the results of the assays could be compared. As observed in Figure 2B, minimal responses to the presence of the analytes was observed despite the high analyte concentration tested (100 µM), compared to the serotonin assay (5 µM). These results confirmed the critical role that the DNA aptamer plays in the assay response and its selectivity.

**Figure 3:**
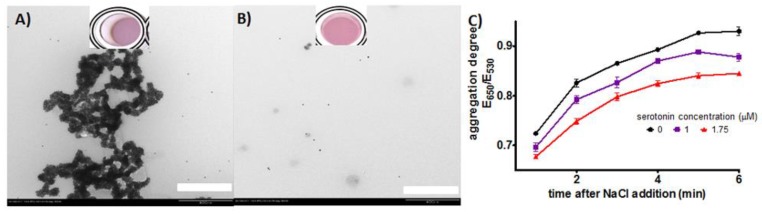
TEM images of Apt-AuNPs exposed to (**A**) blank and (**B**) serotonin (5 µM), followed by addition of NaCl, scale bar 400 nm. (**C**) Kinetics aggregation plot showing the change in aggregation degree after NaCl addition over time, NaCl = 550 mM. Error bars represent the standard deviation of three independent experiments.

To confirm that the response observed due to serotonin binding was a result of Apt-AuNP aggregation, we imaged the system via TEM after addition of a blank solution and serotonin (5 µM) followed by NaCl addition. To avoid over aggregation due to drying, the NaCl concentration was reduced to half the concentration used in the typical assay; furthermore, the liquid was removed after a 15 min incubation in the grid. The images clearly showed that significant AuNP aggregation is observed in the absence of serotonin, but well-dispersed AuNPs are observed after serotonin binding (Figure 3A,B). We acknowledge that the kinetics of the aggregation process could be different than under the typical assay conditions, but it has been shown in the past that this AuNP treatment for TEM imaging correlates qualitatively well with the assay in solution [24]. Apt-AuNP aggregation is a dynamic process that is triggered by NaCl [28]. As shown in Figure 3C, the time allowed for Apt-AuNP aggregation in the presence of the target can be selected to provide the largest difference with the blank and to improve the sensitivity of the system. Importantly, even one minute after salt addition, the difference in response can be observed, showing that a fast response can be obtained with these systems. Based on this data, a 5 min aggregation time was selected before measuring the aggregation to characterize the linear response of the system to serotonin.

### 3.2. Mechanism of Response

**Figure 4:**
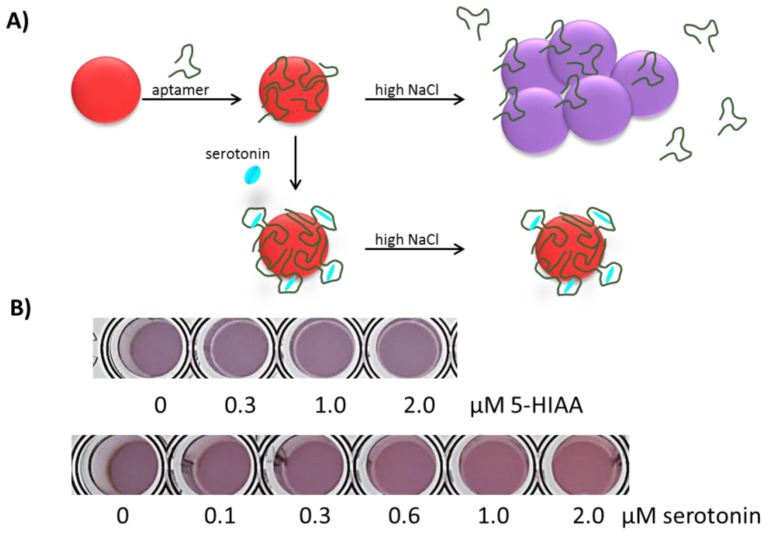
Proposed mechanism of Apt-AuNPs’ response to serotonin. (**A**) Schematic representation of the proposed mechanism, in which target binding results in an aptamer conformation that improves Apt-AuNPs’ stability against salt-induced aggregation. (**B**) Assay color change in response to serotonin and its metabolite 5-HIAA.

Based on the assay characterization data, we propose a response mechanism, as shown in Figure 4, in which serotonin binding to the aptamer resulted in a DNA conformation with higher affinity for the AuNPs’ surfaces. The interaction between the serotonin-aptamer complex and the AuNPs’ surfaces improves the AuNPs’ stability; however, the specific AuNP-aptamer interactions that resulted in this mechanism of response are currently under investigation and are believed to be dependent on the specific conformational change induced by serotonin binding. Importantly, the assay response can be observed with the naked eye, as shown in Figure 4B, which shows that this assay has great potential for interfacing with analytical tools for color quantification developed in portable devices, including cell phones and tablets, as has been recently demonstrated [23].

### 3.3. Serotonin Assay Response Characterization

**Figure 5:**
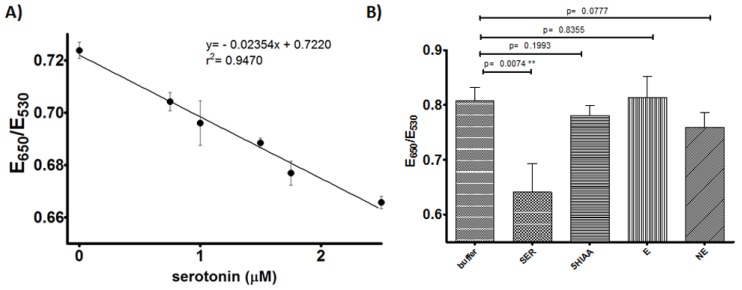
Serotonin assay characterization. (**A**) Assay linear response to serotonin in buffer, NaCl = 450 mM (**B**) Assay response to 600 nM serotonin and 1 µM chemical analogs, NaCl = 374 mM. Error bars represent the standard deviation from three independent experiments.

With the optimized conditions for Apt-AuNPs’ response, we designed a serotonin assay and characterized its response over biologically relevant levels. As shown in Figure 5A, a satisfactory linear response was observed in the physiologically relevant concentration range of 750 nM to 2.5 µM (132 ng/mL to 440 ng/mL), with a limit of detection based on the signal of the blank minus three times its standard deviation of ~300 nM (52 ng/mL). The selectivity of the assay was characterized by comparing the responses to serotonin (600 nM) and its metabolite 5-HIAA (1 µM) (Figure 5B), observing a minimal response to 5-HIAA even at concentrations well above its typical concentration in blood (~100 nM) [10]. Similarly, the assay was tested with other relevant neurotransmitters, epinephrine and norepinephrine (1 µM each), observing responses with no statistical significance. Importantly, the concentrations tested were one order of magnitude higher than typical blood NE values and at least two orders of magnitude higher for E. These data demonstrated the selectivity of the assay and showed promise for serotonin detection in biofluids. We are currently working on addressing the stability of the Apt-AuNPs over time. In general, the response of the assay is maintained for at least a few weeks when stored at 4 °C, but we have observed that, within a few days of the Apt-AuNPs preparation, the overall sensitivity of the assay can be affected. For instance, the data in Figure 5B was obtained using a lower NaCl concentration than 5A, but the aggregation degree was higher since this was run with a fresh batch of AuNPs. As the particles age, the sensitivity to NaCl decreases, but the response to its analyte is maintained. We are currently exploring different storage approaches including lyophilization of the Apt-AuNPs, which has been demonstrated to help maintain Apt-AuNPs’ sensitivity to their targets [31].

### 3.4. Initial Assay Characterization in Biofluids

**Figure 6:**
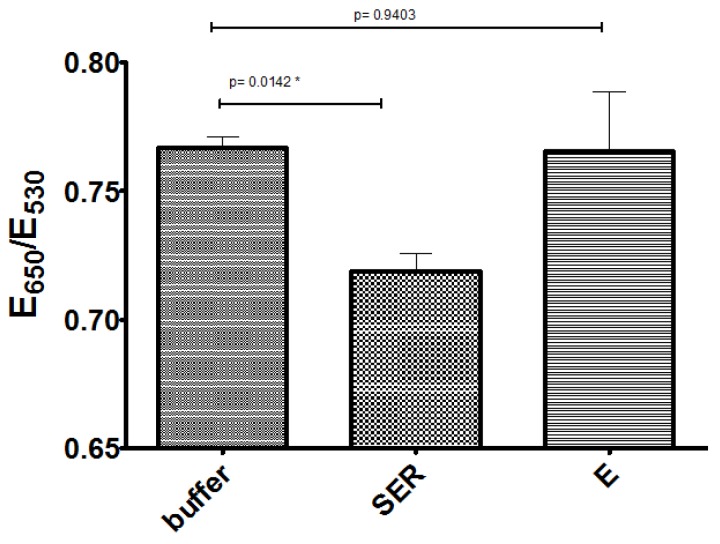
Assay response to 1.5 µM analytes in treated-fetal bovine serum (FBS). Samples were diluted from a buffer stock solution to a final treated-FBS content of 75% (NaCl = 690 mM). Error bars represent the standard deviation from two independent experiments.

Finally, we tested the performance of these Apt-AuNPs in spiked fetal bovine serum (FBS), as a mimic of biological samples. In this case, we could not use 5-HIAA as a negative control, since we found that the stock methanol solutions of serotonin and 5-HIAA further diluted with FBS resulted in inconsistent results (the reason for these findings are still under investigation). Therefore, stock solutions of the hydrochloride salts of serotonin and epinephrine were prepared in a buffer and finally diluted with FBS. The absence of methanol in the FBS samples seemed to prevent the issues found in the preliminary experiments. The samples were treated simply by spinning FBS through Amicon filters to remove proteins present in these biofluids, which was observed in preliminary experiments to prevent the Apt-AuNPs’ response to serotonin. It was noticeable that the presence of the proteins prevented the Apt-AuNP aggregation even at high NaCl concentrations. We hypothesized that electrostatic-driven protein adsorption on the negatively charged Apt-AuNPs resulted in surface passivation that masked the serotonin binding effect on the Apt-AuNPs’ stability. Accordingly, we observed that the NaCl concentration needed to promote the assay response was significantly higher than that in the buffer (690 mM). Importantly, spiked samples with a final FBS content of 75% showed the expected response to 1.5 µM serotonin with no statistically significant response to epinephrine, showing the robust nature of these Apt-AuNPs (Figure 6). These findings suggest that minimal sample treatment/dilution might be necessary to quantify serotonin in biofluids, confirming that these Apt-AuNPs conjugates are promising biosensors for fast biomarker detection.

## 4. Conclusions

Aptamer-AuNP conjugates were designed by adsorbing an aptamer to citrate-stabilized AuNPs. It was observed that the Apt-AuNPs improved their stability against salt-induced aggregation as a consequence of serotonin binding. A mechanism of response in which an analyte binding to the aptamer resulted in a target-DNA complex that remained on the AuNPs’ surfaces and improved the AuNPs’ stability was proposed and is currently under investigation. A plasmonic assay that responded to serotonin in biologically relevant levels was designed based on these Apt-AuNPs. Importantly, the assay involved simply mixing the samples with the Apt-AuNPs and, after a 5 min incubation time, with the addition of NaCl with a total assay time of less than 15 min. The added stability of the analyte-bound conjugates allowed for the use of the serotonin assay in biofluids, opening the door for their use in different applications including clinical biomarker quantification and point-of-care diagnostics.
